# The interplay of the Notch signaling in hepatic stellate cells and macrophages determines the fate of liver fibrogenesis

**DOI:** 10.1038/srep18272

**Published:** 2015-12-14

**Authors:** Ruchi Bansal, Joop van Baarlen, Gert Storm, Jai Prakash

**Affiliations:** 1 Targeted Therapeutics, Department of Biomaterials Science and Technology, MIRA Institute for Biomedical Technology and Technical Medicine, Faculty of Science and Technology, University of Twente, Enschede, 7522NB, The Netherlands; 2 Laboratorium Pathologie Oost-Nederland, Hengelo, 7555 BB, The Netherlands; 3 Department of Pharmaceutics, Utrecht Institute for Pharmaceutical Sciences, Faculty of Science, Utrecht University, Utrecht, 3584 CG, The Netherlands

## Abstract

Hepatic stellate cells (HSCs) known as “master producers” and macrophages as “master regulators”, are the key cell types that strongly contribute to the progression of liver fibrosis. Since Notch signaling regulates multiple cellular processes, we aimed to study the role of Notch signaling in HSCs differentiation and macrophages polarization and to evaluate its implication in liver fibrogenesis. Notch pathway components were found to be significantly upregulated in TGFβ-activated HSCs, inflammatory M1 macrophages, and in mouse and human fibrotic livers. Interestingly, inhibition of Notch using a selective γ-secretase inhibitor, Avagacestat, significantly inhibited TGFβ-induced HSC activation and contractility, and suppressed M1 macrophages. Additionally, Avagacestat inhibited M1 driven-fibroblasts activation and fibroblasts-driven M1 polarization (nitric oxide release) in fibroblasts and macrophages co-culture, and conditioned medium studies. *In vivo*, post-disease treatment with Avagacestat significantly attenuated fibrogenesis in CCl_4_-induced liver fibrosis mouse model. These effects were attributed to the reduction in HSCs activation, and inhibition of inflammatory M1 macrophages and upregulation of suppressive M2 macrophages. These findings suggest that Notch signaling plays a crucial role in HSC activation and M1/M2 polarization of macrophages in liver fibrosis. These results provide new insights for the development of novel therapies against liver fibrosis through modulation of Notch signaling.

Hepatic fibrosis, characterized by excessive accumulation of abnormal extracellular matrix (ECM) proteins leading to liver dysfunction, is a growing cause of mortality worldwide^1^,^2^,^3^. Despite our increasing understanding of cellular and molecular mechanisms contributing to liver fibrosis, there are no effective and clinically approved anti-fibrotic therapies available^4^,^5^. Hepatocellular damage due to liver injury leads to the release of pro-fibrotic factors from infiltrating inflammatory cells especially macrophages, resulting in the activation of quiescent hepatic stellate cells (HSCs). Upon activation, HSCs undergo characteristic morphological and functional changes, and are transformed into proliferative and contractile ECM-producing myofibroblasts^6^,^7^.

Macrophages are found in close proximity of the activated HSCs and indisputably play a key role in fibrosis initiation and progression^8^,^9^. Hepatic macrophages can arise either from circulating bone-marrow derived monocytes, which are recruited to the injured liver, or from proliferating resident macrophages (Kupffer cells). Resident macrophages have shown to play a role in initiating inflammatory responses during tissue injury, while infiltrating monocyte-derived macrophages leads to chronic liver inflammation and fibrogenesis^8^,^10^,^11^. During liver damage, both resident and recruited macrophages produces pro-fibrotic mediators such as transforming growth factor (TGFβ) and platelet-derived growth factor (PDGF), cytokines and chemokines that activate fibroblasts and recruit circulating monocytes and other inflammatory cells^8^,^10^,^11^. During enhanced recruitment owing to liver damage and environmental cues, infiltrating monocytes undergo differentiation into two broad subsets of macrophages that are categorized as classically-activated (M1) or alternatively-activated (M2). The initial inflammatory response is predominantly mediated by classically-activated (M1-differentiated) macrophages (activated by Th1 cytokines e.g. IFN-γ, LPS, TNF-α or IL-12)^10^,^11^,^12^. In contrast, the resolution phase of inflammation is driven by alternatively-activated (M2-differentiated) macrophages, stimulated by Th2 cytokines IL-4 or IL-13^10^,^11^,^12^. Therefore, HSCs known as “master producers” and macrophages (inflammatory cells) as “master regulators” of fibrosis, represents the key cell types that strongly contribute to the initiation and progression of liver fibrosis^2^,^13^.

During fetal development and liver regeneration, several highly conserved signaling pathways (Wnt, Hedgehog and Notch) are utilized that orchestrate organogenesis^14^. One of these pathways is the Notch pathway, which has been shown to be aberrantly upregulated during several malignancies and fibrotic diseases^15^,^16^,^17^,^18^,^19^. Notch signaling pathway plays an essential role in a number of processes during embryonic development including cardiogenesis, vasculo-angiogenesis, hematopoiesis and neurogenesis. These processes involves stem cell self-renewal, proliferation, cell fate determination, and apoptosis^20^,^21^. In mammals, four transmembrane Notch receptors (Notch-1, -2, -3 and -4) and two types of ligands, Jagged (Jag-1 and -2) or Delta-like (Dll-1, -3 and -4) have been identified^20^,^21^. Interaction between Notch receptor- and Notch ligand-expressing cells leads to the activation of Notch signaling cascade resulting in the cleavage of ligand-activated Notch receptor by γ-secretase complex^21^. The cleaved Notch intracellular domain (NICD) translocate into the nucleus, where it binds and activates DNA-binding recombination signal-binding protein Jκ (RBP-Jκ) resulting in transcription of Notch target genes e.g. hairy/enhancer of split 1 (Hes1)^21^.

In this study, we demonstrate that Notch signaling pathway plays a dual role by regulating HSC activation/differentiation and in determining M1 versus M2 polarization *in vitro* and *in vivo* in liver fibrosis. Notch signaling pathway was upregulated in activated hepatic stellate cells and inflammatory M1 macrophages suggesting Notch-mediated regulation during fibrosis progression. We therefore hypothesized that inhibition of this pathway at the fibrogenic stage might alleviate the fibrosis progression by inhibiting HSC activation and macrophage polarization.

## Results

### Notch signaling pathway in mouse and human cirrhotic livers

**Figure 1:**
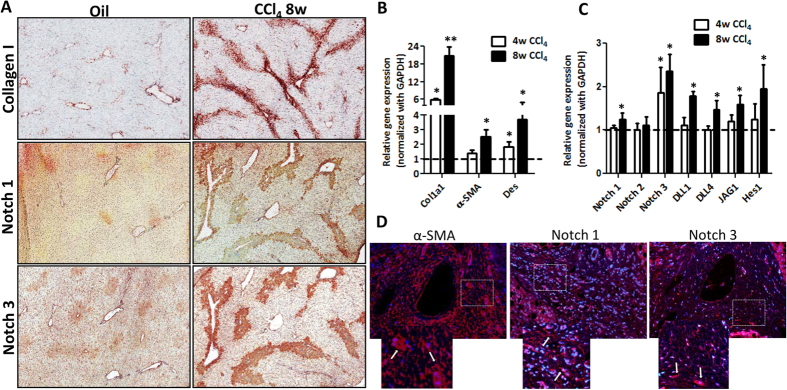
Notch signaling pathway in CCl_4_-induced chronic liver fibrosis in mice and fibrotic human livers. (**A**) Representative photomicrographs (200 μm) showing Collagen I, Notch-1 and Notch-3 stained liver sections from olive oil-treated (non-fibrotic) control mice and CCl_4_-treated (fibrotic) mice (n = 5). Quantitative gene expression (normalized with GAPDH) of (**B**) fibrotic parameters (Col1A1, α-SMA and desmin), and (**C**) Notch pathway components: Notch receptors (Notch-1, -2 and -3), Notch ligands (Dll1, Dll4 and Jag1) and downstream Notch signaling molecule (Hes1) in the livers of olive oil-treated (non-fibrotic controls) and CCl_4_-treated (fibrotic) mice. (**D**) Representative fluorescent photomicrographs (200 μm) showing α-SMA, Notch-1 and Notch-3 stained liver sections (in red) from fibrotic human livers (n = 4). Nuclei were stained blue using DAPI. The lower panel shows a zoomed in area, where white arrows indicates positive staining. Bars represent mean ± SEM of n = 5. *p < 0.05 and **p < 0.01 denotes significance versus respective olive oil treated control group (denoted as dotted line).

To study the role of Notch signaling pathway in liver fibrosis, we first investigated the hepatic expression of Notch pathway related genes in progressive CCl_4_-induced liver fibrosis mouse models (4 weeks and 8 weeks) as compared to the olive oil treated non-fibrotic control group. CCl_4_-treated fibrotic mice developed extensive bridging fibrosis, substantial deposition of collagen I and increased expression of HSC markers, α-SMA and desmin (Fig. 1A,B). As shown in Fig. 1A,C, both protein and gene expression of Notch receptors (Notch-1 and -3) were significantly upregulated after 8 weeks of liver fibrosis. Furthermore, Notch ligands (Dll1, Dll4, Jag1) and downstream molecule (Hes1) were also significantly induced in fibrotic mice versus olive-oil treated non-fibrotic control livers (Fig. 1C) signifying the role of Notch pathway in liver fibrosis. In addition, we found the increased expression of Notch receptors, Notch-1 and -3 was localized in the fibrous region i.e. α-SMA positive areas (as shown with white arrows in the zoomed images) in the cirrhotic livers (Fig. 1D).

### Notch signaling in activated human hepatic stellate cells and macrophages

**Figure 2:**
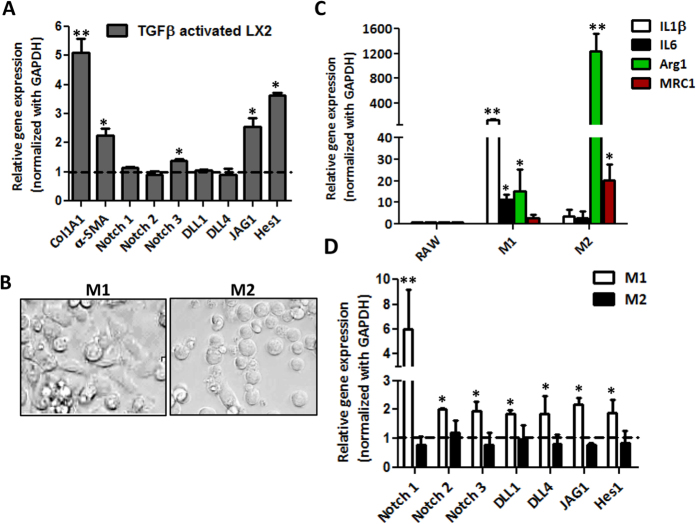
Expression of Notch signaling pathway activation in human HSCs and macrophages. (**A**) Quantitative gene expression analysis of fibrotic marker (Col1A1), HSC activation marker (α-SMA) Notch receptors (Notch-1,-2 and -3), Notch ligands (Dll1, Dll4 and Jag1) and downstream Notch signaling molecule (Hes1) in TGFβ-activated HSCs versus control HSCs (expressed as dotted line). (**B**) Representative microscopic images of M1-differentiated (LPS/IFNγ treated) and M2-differentiated (IL-4/IL-13 treated) macrophages (scale bars, 200 μm). Quantitative gene expression analysis of (**C**) M1-specific markers (IL-1β and IL-6) and M2-specific markers (Arginase I, Arg1 and Mannose receptor C type 1, MRC1); (**D**) Notch pathway related genes: Notch receptors (Notch-1, -2 and -3); Notch ligands (Dll1, Dll4 and Jag1) and downstream Notch signaling molecule (Hes1), in M1-differentiated and M2-differentiated RAW macrophages. Untreated/undifferentiated RAW cells are denoted as dotted line. Bars represent mean ± SEM of atleast three independent experiments. *p < 0.05 and **p < 0.01 denotes significance versus respective control cells.

Since HSCs and macrophages are the key cells in the fibrous areas^22^, we investigated the activation of Notch pathway activation in these cells *in vitro*. In TGFβ-activated human HSCs, we found the expression of fibrosis-related parameters (collagen I and α-SMA), and Notch pathway related genes (Notch3, Jag1 and Hes1) were significantly upregulated as compared to non-activated control cells (Fig. 2A).

To examine the Notch signaling pathway in macrophages, we differentiated mouse macrophages to M1 or M2 macrophages using LPS and IFNγ (for M1) or IL-4 and IL-13 (for M2). As shown in Fig. 2B, the polarization to M1 and M2 macrophages exhibited morphological differences. After stimulation with LPS/IFNγ, M1 macrophages preferentially showed long and spindle-shaped morphology while IL4/IL13 (M2) differentiated macrophages were largely round-shaped (Fig. 2B). The M1 and M2 phenotypes were further confirmed by gene expression analysis. As compared to undifferentiated RAW cells, M1-differentiated macrophages showed significant upregulation of M1-specific inflammatory genes (IL-1β and IL-6) while M2-specific genes (Arginase 1 and Mannose receptor C type 1, MRC1) were substantially induced in M2-differentiated macrophages (Fig. 2C).

Thereafter, we investigated Notch signaling pathway related genes in RAW, M1- and M2-differentiated macrophages. We found that M1 macrophages expressed higher levels of Notch receptors (Notch1 > Notch3 > Notch2) and ligands (Dll1, Dll4, Jag1) and the downstream signaling molecule Hes1 as compared to un-differentiated or M2 macrophages. These data suggest that Notch pathway is strongly activated in M1-differentiated macrophages in comparison to un-differentiated and M2-differentiated macrophages (Fig. 2D).

### Notch inhibitor Avagacestat inhibits differentiation and contractility of HSCs

**Figure 3:**
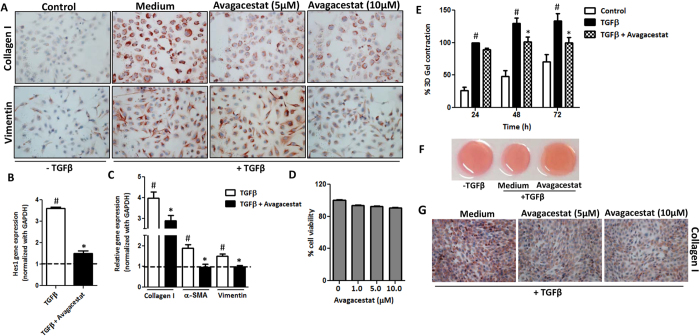
Effect of Notch inhibition on the activation and contractility of TGFβ-activated human HSCs. (**A**) Representative images (scale bars, 200 μm) of collagen I and vimentin stained LX2 cells treated with medium alone (control), TGFβ (5 ng/ml) ± 5 μM or 10 μM Notch inhibitor (Avagacestat). Quantitative gene expression analysis (normalized with GAPDH) of (**B**) Notch signaling molecule Hes1, (**C**) Fibrotic parameters: collagen I, α-SMA and Vimentin on LX2 cells treated with medium alone (control expressed as dotted line), TGFβ (5 ng/ml) ± 10 μM Avagacestat. (**D**) Graph showing % cell viability performed on LX2 cells incubated with increasing concentrations of Avagacestat. (**E**) Graph depicts % 3D-collagen-I gel contraction after 24, 48 and 72 h of treatment with medium alone (control), TGFβ (5 ng/ml) ± 10 μM Avagacestat. (**F**) Representative microscopic images of the contracted collagen gels after 72 h of treatments. (**G**) Representative pictures (scale bars, 200 μm) of collagen I stained mouse 3T3 fibroblasts treated with medium alone (control), TGFβ (5 ng/ml) ± 5 μM or 10 μM Avagacestat. Bars represent mean ± SEM of atleast three independent experiments. ^#^p < 0.05 denotes significance versus respective control cells; *p < 0.05 denotes significance versus TGFβ-treated cells.

To understand the role of Notch signaling pathway in fibrogenic cells, we inhibited Notch signaling pathway in human HSCs using a selective arylsulfonamide γ-secretase Notch inhibitor, Avagacestat. We observed that following TGFβ activation, there was a significant increase in protein and gene expression of fibrotic parameters (collagen-I, α-SMA and vimentin) (Fig. 3A,C). Inhibition of Notch signaling pathway with Avagacestat, which was confirmed by significant decrease in Hes1 expression (Fig. 3B), led to the dose-dependent reduction in collagen-I, α-SMA and vimentin protein expression and also a significant inhibition in the respective mRNA expression levels was observed (Fig. 3A,C). No significant effect on cell viability was found at different concentrations of Avagacestat, indicating no effect on cell death or proliferation (Fig. 3D).

Furthermore, we examined the effect of Avagacestat on the contractility of human HSCs in 3D collagen gel. We found that 10 μM Avagacestat significantly inhibited TGFβ-induced contractility of HSCs after 48 h and 72 h as shown in Fig. 3E,F. In addition, Avagacestat showed inhibition of collagen-I expression in activated mouse 3T3 fibroblasts (Fig. 3G) indicating its inhibitory effect in both mouse and human fibroblasts.

### Blocking of Notch signaling inhibits M1-polarization

**Figure 4:**
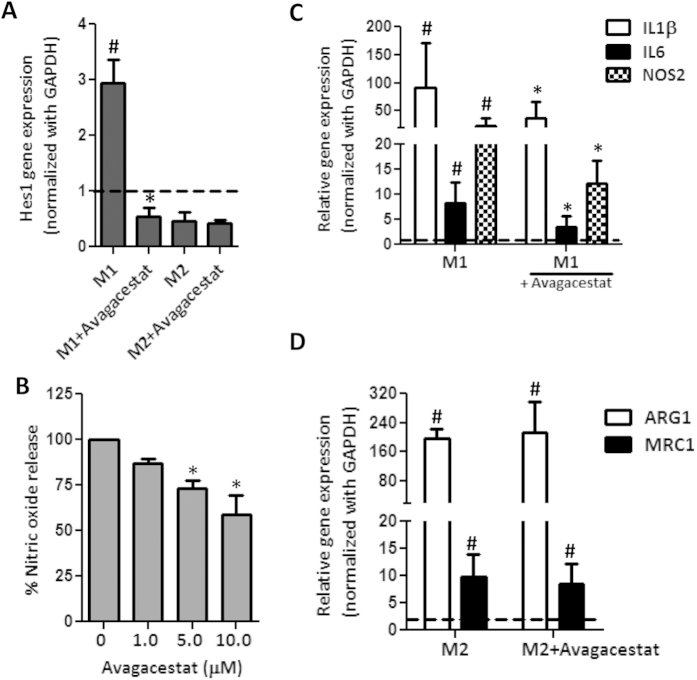
Effect of Notch inhibition on the polarization of M1- and M2-differentiated macrophages. (**A**) Quantitative gene expression analysis of Hes1 in M1-differentiated and M2-differentiated RAW macrophages incubated with 10 μM Avagacestat. Untreated/undifferentiated RAW macrophages are expressed as dotted line. (**B**) Graph depicts nitric oxide release (expressed in %) by M1-differentiated macrophages incubated with increasing concentrations of Avagacestat. (**C**) Quantitative gene expression analysis of M1 markers (IL-1β, IL-6 and NOS2) in M1-differentiated RAW macrophages incubated with 10 μM Avagacestat. (**D**) Quantitative gene expression analysis of M2 markers (Arg1 and MRC1) in M2-differentiated RAW macrophages incubated with 10 μM Avagacestat. Untreated/undifferentiated RAW macrophages are expressed as dotted line. Bars represent mean ± SEM of atleast three independent experiments. ^#^p < 0.05 denotes significance versus respective untreated RAW cells. *p < 0.05 denotes significance versus M1-differentiated RAW macrophages.

We then investigated the role of Notch signaling in macrophage polarization to M1 and M2. We found that incubation with the Notch inhibitor, Avagacestat completely inhibited the M1-specific Notch signaling gene Hes1 (Fig. 4A). Furthermore, treatment with Avagacestat dose-dependently inhibited the M1-induced nitric oxide (NO) release (an indicator of M1 activation) (Fig. 4B). Of note, at these doses, there was no effect on the cell viability of macrophages (data not shown). We further found that Avagacestat led to the substantially reduced M1 polarization as depicted in M1-related genes i.e IL-1β, IL-6 and nitric oxide synthase 2 (NOS2) (Fig. 4C). No differences in M2 polarization were observed following treatment with the Notch inhibitor, as shown with mRNA expression analyses (Fig. 4D).

### Effect of Avagacestat on the crosstalk between macrophages and fibroblasts

**Figure 5:**
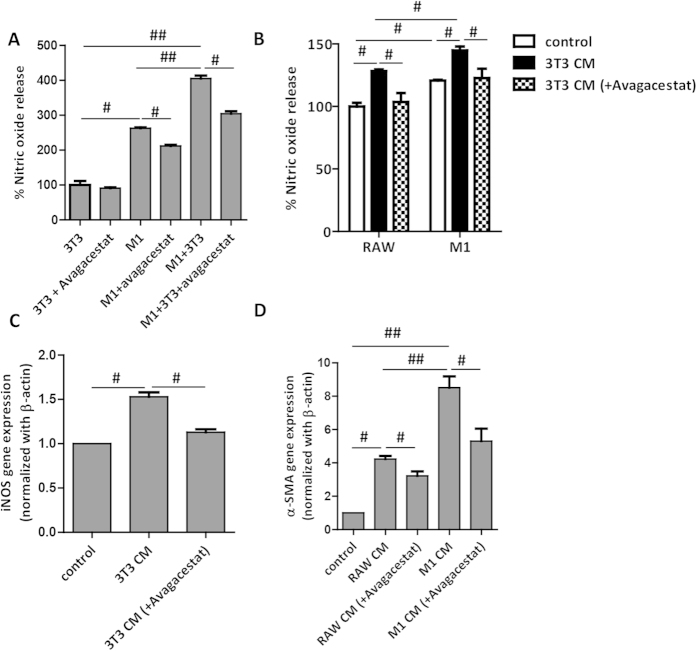
Effect of Avagacestat on the cross-talk between 3T3 fibroblasts and RAW macrophages. (**A**) Graph depicting nitric oxide release (expressed in %) from RAW macrophages and 3T3 fibroblasts cultured either alone or together (co-culture). 3T3 (TGFβ-activated) and M1-differentiated macrophages were treated with 10 μM Avagacestat either separately or in co-culture. (**B**) Graph depicts nitric oxide release (expressed in %) from undifferentiated RAW macrophages and M1-differentiated macrophages treated with medium alone (control), 3T3-conditioned medium (3T3 CM) and Avagacestat (10 μM) treated 3T3-conditioned medium (3T3 CM + Avagacestat). (**C**) Quantitative gene expression analysis of iNOS (normalized with GAPDH) in RAW macrophages treated with medium alone (control), 3T3-conditioned medium (3T3 CM) and Avagacestat (10 μM) treated 3T3-conditioned medium (3T3 CM + Avagacestat). (**D**) α-SMA gene expression analysis (normalized with GADPH) in 3T3 fibroblasts treated with medium alone (control), undifferentiated RAW macrophages conditioned medium (RAW CM), Avagacestat (10 μM) treated RAW macrophages conditioned medium (RAW CM + Avagacestat), M1-differentiated macrophages conditioned medium (M1 CM) and Avagacestat (10 μM) treated M1-differentiated macrophages conditioned medium (M1 CM + Avagacestat). Bars represent mean ± SEM of atleast three independent experiments. ^#^p < 0.05, ^##^p < 0.01 denotes significance.

Since fibrosis is driven by the cross-talk between macrophages and fibroblasts (or activated HSCs), we designed experiments to determine the effect of Avagacestat on the interplay between macrophages and fibroblasts. We performed co-culture studies using mouse 3T3 fibroblasts and mouse RAW macrophages, and analyzed nitric oxide release. We found that co-culture of 3T3 and RAW macrophages alone (without TGFβ and M1 cytokines LPS and IFNγ) did not induce nitric oxide release (data not shown), but treatment with TGFβ (3T3 activation) and LPS/IFNγ (M1-differentiation) led to highly significant increase in NO release as compared to M1 macrophages mono-culture suggesting activated fibroblasts stimulates M1 activation. This 3T3-driven M1 activation was significantly down-regulated by Notch Inhibitor, Avagacestat (Fig. 5A). In co-culture studies, it might be possible that Avagacestat inhibited both M1 macrophages differentiation and 3T3 activation. Furthermore, 3T3-driven M1 activation may have resulted from direct contact and/or secreted fibroblast factors.

To determine whether macrophage can activate 3T3 fibroblasts or vice versa, we established conditioned medium studies. Undifferentiated RAW cells and M1-differentiated macrophages were treated with medium alone, conditioned medium from 3T3 fibroblasts or Avagacestat-treated 3T3 fibroblasts. We found that 3T3 conditioned medium alone stimulated M1-differentiation in undifferentiated RAW macrophages and further activated M1-differentiated macrophages as analyzed by NO release (Fig. 5B). This fibroblasts-driven macrophage activation was blunted when Avagacestat-treated fibroblast conditioned medium was added on macrophages (both undifferentiated and M1-differentiated) suggesting role of Notch pathway in 3T3-driven macrophage activation. These results were further confirmed by quantitative PCR where 3T3 conditioned medium stimulated iNOS gene expression, which was inhibited by Avagacestat-treated 3T3 conditioned medium (Fig. 5C).

On the other hand, we determined effect of RAW macrophages or M1-differentiated macrophages on 3T3 fibroblasts activation. Undifferentiated macrophage-conditioned medium alone induced 3T3 activation as analyzed by α-SMA gene expression (Fig. 5D). However, M1-differentiated macrophage-conditioned medium induced drastic (>2-fold higher) activation of fibroblasts suggesting role of macrophages and M1-macrophages in fibroblasts activation. This macrophage-driven fibroblasts activation was inhibited by Avagacestat-treated macrophage conditioned medium suggesting role of Notch pathway in 3T3 activation (Fig. 5D).

These co-culture and conditioned medium studies demonstrate cross-talk between macrophages and fibroblasts via direct contact (juxtacrine effect) and/or via secreted stimulating factors (non-contact/paracrine effect) and suggests an important regulatory role of Notch signaling in this cross-talk or interplay.

### Effect of Avagacestat in acute liver injury mouse model

**Figure 6:**
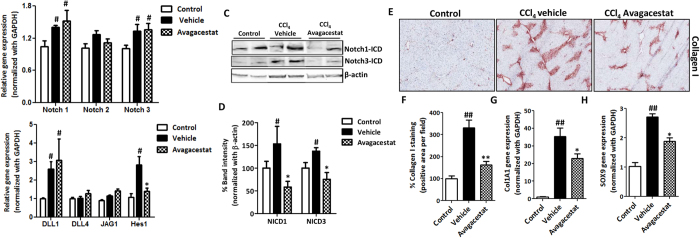
Effect of Notch pathway inhibition on CCl_4_-induced acute liver fibrogenesis. Quantitative real time PCR analysis for (**A**) Notch receptors (Notch-1, -2 and -3); (**B**) Notch ligands (Dll1, Dll4 and Jag1) and Notch signaling molecule Hes1 in olive oil-treated control, vehicle- and Avagacestat-treated CCl_4_ mice. Representative image (**C**) and quantitative analysis (**D**) of the Western blots for Notch-1 intracellular domain (NICD1), Notch-3 intracellular domain (NICD3) and β-actin performed on liver homogenates from olive oil-treated control, vehicle- and Avagacestat-treated CCl_4_ mice. Each band represents an individual mice. Quantitation was performed in n = 3 mice per group and represented as averaged % band intensity (normalized with respective β-actin band intensity). (**E**) Representative photomicrographs (200 μm) and (**F**) Quantitative histological analysis of Collagen I stained liver sections from olive oil-treated (control), vehicle- and Avagacestat-treated CCl_4_ mice. Quantitative gene expression analysis of (**G**) collagen I (Col1A1) and (**H**) Sox9 in the livers of different treated groups. Bars represent mean ± SEM of n = 5. ^#^p < 0.05, ^##^p < 0.01 denotes significance versus respective olive-oil treated control group; *p < 0.05, **p < 0.01 denotes significance versus CCl_4_-treated vehicle group.

The effect of Avagacestat on early fibrogenesis was assessed in acute liver injury model in mice. The Notch pathway was found to be upregulated in the short-term CCl_4_-induced liver injury mouse model as depicted by the induction of Notch pathway related genes in CCl_4_-treated mice as compared to the olive-oil treated control mice (Fig. 6A,B). Since Avagacestat inhibits downstream Notch signaling pathway (via inhibition of NICD cleavage/release thereby preventing NICD nuclear translocation and Notch target genes expression), *in vivo* inhibition with Avagacestat was confirmed by down-regulation of Notch target gene (Hes1) expression (Fig. 6B). We further analyzed the NICD1 and NICD3 translocation/cleavage via western blot analysis. We found that NICD1 and NICD3 expression was increased in fibrotic mice as compared to the olive-oil treated control mice. Interestingly, Avagacestat treatment significantly inhibited NICD1 and NICD3 protein expression or translocation (Fig. 6C,D). As expected, Avagacestat did not induce any changes in gene expression levels of upstream Notch receptors (Notch-1, -2 and -3) and ligands (Dll1, Dll4 and Jag1), both *in vitro* (data not shown) and *in vivo* (Fig. 6A,B).

Interestingly, Avagacestat administration induced a strong inhibition of early fibrosis as shown by the significant decrease in collagen-I protein and mRNA expression levels in Avagacestat-treated mice as compared to untreated mice (Fig. 6E–G). In addition, we observed that SRY (sex determining region Y)-box 9 (SOX9), that regulates collagen expression during organ fibrogenesis, that is also regulated by Notch signaling pathway^23^,^24^, was significantly increased in fibrotic mice as compared to control mice. Interestingly, Avagacestat significantly inhibited SOX9 expression suggesting Notch regulates SOX9 expression and collagen-I expression in liver fibrosis (Fig. 6H).

**Figure 7:**
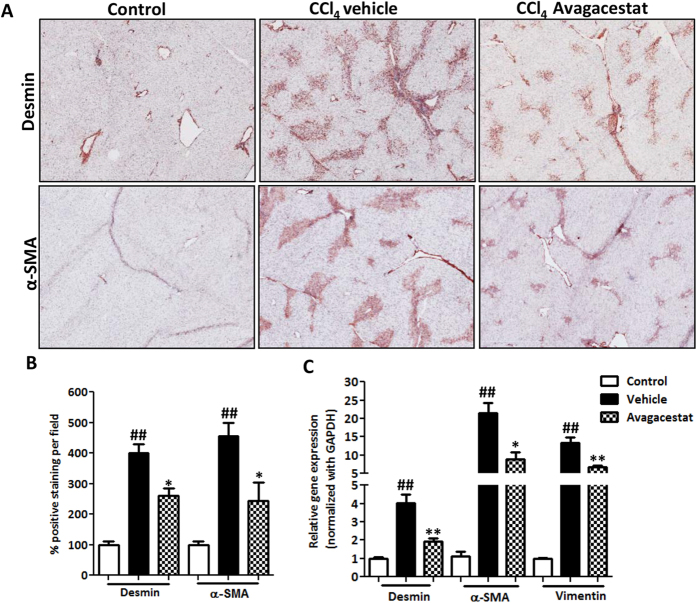
Effect of Notch inhibition on HSC activation in CCl_4_-induced acute liver injury. (**A**) Representative photomicrographs (200 μm) and (**B**) quantitative histological analysis of α-SMA and Desmin stained liver sections from olive oil-treated (control), vehicle- and Avagacestat-treated CCl_4_ mice. (**C**) Quantitative gene expression analysis of α-SMA, Desmin and Vimentin in the livers of different treated groups. Bars represent mean ± SEM of n = 5. ^##^p < 0.01 denotes significance versus respective olive-oil treated control group; *p < 0.05, **p < 0.01 denotes significance versus CCl_4_-treated vehicle group.

We further investigated the effect of Avagacestat in HSCs activation *in vivo*. We found that Avagacestat treatment induced a significant reduction in the CCl_4_-induced expression of HSCs-specific markers, α-SMA and desmin (Fig. 7A,B). These reductions in immuno-stainings were paralleled by the corresponding reductions in mRNA gene expression levels of α-SMA, Desmin and Vimentin (Fig. 7C).

**Figure 8:**
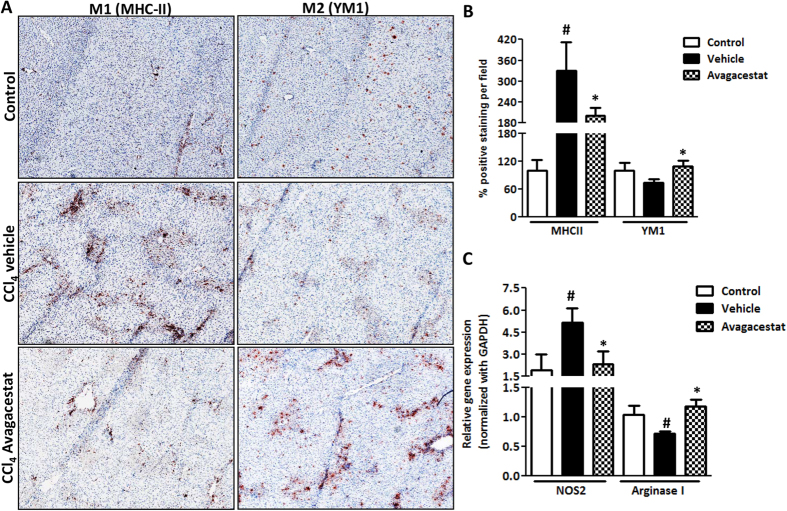
Effect of Avagacestat on macrophage polarization in CCl_4_-induced early liver fibrosis. (**A**) Representative photomicrographs (200 μm) and (**B**) quantitative histological analysis of MHC-II and YM1 stained liver sections from olive oil-treated (control), vehicle- and Avagacestat-treated CCl_4_ mice. (**C**) Quantitative gene expression analysis of NOS2 and Arginase I in the livers of different treated groups. Bars represent mean ± SEM of n = 5. ^#^p < 0.05 denotes significance versus respective olive oil treated control group; *p < 0.05 denotes significance versus CCl_4_-treated vehicle group.

To study whether the effects of Avagacestat on fibrosis were related to the changes in the macrophages, we investigated the status of M1 and M2 macrophages in the control and Avagacestat-treated livers. As shown in Fig. 8A,B, we found that Avagacestat suppressed M1 macrophage polarization as confirmed with MHC-II immunostaining (an inflammatory marker) but induced M2 macrophage polarization, as confirmed by YM1 staining (M2-specific marker) (Fig. 8A,B). The M1 and M2 skewing was further confirmed with quantitative PCR for selective M1 (NOS2) and M2 (Arginase I) markers, further illustrating down-regulation of M1 macrophages while upregulation of M2 macrophages (Fig. 8C).

## Discussion

In this study, we unveil the dual role of Notch signaling in HSC activation and in determining M1 versus M2 polarization *in vitro* and *in vivo* in liver fibrosis. We demonstrate the activation of Notch pathway in human fibrotic livers and CCl_4_-induced chronic and progressive liver fibrosis in mice. Furthermore, we show differential upregulation of Notch receptors and ligands in TGFβ-activated HSCs and in M1-differentiated inflammatory macrophages. Inhibition of Notch signaling using a γ-secretase inhibitor Avagacestat, strongly suppressed HSC activation, collagen deposition and HSC contractility *in vitro*. Additionally, blocking of Notch signaling inhibited M1 polarization in differentiated macrophages. Interestingly, Avagacestat significantly inhibited M1 driven-fibroblasts activation and fibroblasts-driven M1 polarization. *In vivo* in CCl_4_-induced early liver fibrogenesis in mice, Notch inhibitor significantly inhibited collagen deposition and HSC differentiation. Remarkably, Avagacestat suppressed inflammatory M1 macrophages while promoted suppressive M2 macrophages. These results suggest a crucial role of Notch signaling in the activated HSCs and inflammatory M1 macrophages, key pathogenic cell types, in liver fibrosis signifying interplay of Notch signaling pathway in determining the pathogenesis of liver fibrosis.

Fibrogenesis is a chronic wound healing response which is characterized by a tight interplay between inflammatory and matrix-producing cellular pathways^1^. Hepatic stellate cells (HSCs) are considered as the major producers of fibrotic ECM during liver fibrosis, but activation of macrophages is also an essential step in the initiation of fibrogenesis and they are signified as positive modulators of fibrosis^25^,^26^,^27^. Owing to liver fibrogenesis, quiescent HSCs become activated to a contractile myofibroblast-like matrix-secreting phenotype and secrete fibrillar collagens resulting in deposition of fibrotic matrix and scar-tissue formation^6^. Therefore, modulation or inhibition of HSC activation is a promising approach to treat fibrotic diseases^28^. Additionally, hepatic immunological environment determines the functional plasticity of macrophages varied from classical inflammatory M1 macrophages to alternatively differentiated restorative M2 macrophages^12^,^29^. Altogether, dysregulation of the M1/M2 phenotypic balance governs the pathogenesis of inflammatory diseases like liver fibrosis suggesting strategies restraining M1 macrophage polarization and/or favoring M2 macrophage phenotype may protect against exacerbated inflammation and thus limit tissue injury.

Notch signaling is increasingly recognized as a major signaling mechanism in liver biology and in multiple pathological conditions from liver damage to carcinogenesis^30^. During liver fibrosis, expression of Notch-3 and Jagged1 (mainly related to HSC activation) has been found to be upregulated in diseased human livers and in animal models^31^,^32^. In line of these studies, we, in the present study confirmed that Notch pathway was activated in moderate to advanced liver fibrosis in mice and human cirrhotic livers. Some studies have shown enhanced M1 gene expression and pro-inflammatory responses owing to the activation of Notch pathway^33^,^34^. However the dual role of Notch signaling pathway in macrophage polarization and HSC activation, and its effects upon inhibition, during liver fibrosis remain elusive. In this study, we sought to delineate the differential Notch expression in activated stellate cells and differentiated macrophages. In accordance with others^31^,^32^, we also found the activation of Notch pathway in TGFβ-activated HSCs and showed that pharmacological inhibition of Notch signaling pathway using Avagacestat attenuated HSCs activation, collagen deposition and their contractility suggesting the crucial role of Notch pathway in the differentiation and activation of HSCs.

Previously, it has been shown that Notch signaling is critically involved in LPS-induced macrophage activation and macrophage plasticity in cancer and autoimmunity^34^,^35^,^36^,^37^. Although, these results suggest that Notch plays a role in the modulation of immune responses but its direct role in liver fibrosis and its therapeutic significance in relation to immune cells remain unanswered. In this study, we have demonstrated the upregulation of Notch signaling pathway in M1-differentiated macrophages, and that pharmacological inhibition of Notch signaling pathway using Avagacestat strongly inhibited M1 phenotype without affecting M2-differentiated macrophages. These data clearly indicated the significance of Notch pathway in both HSCs and M1 macrophages *in vitro*.

Since fibrogenesis is a dynamic process tightly regulated by macrophage-stellate cells interaction^38^,^39^. Cross-talk between macrophages and fibroblasts (or stellate cells) drives fibrogenic process via juxtacrine (cell-cell contact) or paracrine (non-cell contact) signaling pathways^40^. In this study, using co-culture and conditioned medium studies, we demonstrated that 3T3 fibroblasts drives activation of M1-macrophages, and macrophages (and M1-macrophages) drives 3T3 activation. This cross-talk/interplay between fibroblast and macrophages is regulated by Notch signaling pathway, since inhibition of Notch pathway inhibited 3T3-driven macrophage activation/differentiation and macrophage-driven fibroblasts activation.

Furthermore, *in vivo* in CCl_4_-induced early hepatic fibrogenesis mouse model, we showed that the Notch pathway is activated at a very early stage of liver fibrogenesis as confirmed by induction of Notch receptors, ligands and downstream gene. Most importantly, treatment with Avagacestat ameliorated the liver damage which was attributed to reduced HSCs activation and proliferation, and reduction of M1 but induction of M2 macrophages.

In conclusion, this study demonstrates the role of Notch pathway in the interplay of two crucial cell types i.e. stellate cells and inflammatory M1 macrophages in liver fibrosis. Furthermore, it signifies the therapeutic significance of the Notch pathway in liver fibrosis which could lead to development of novel therapies against liver fibrosis. Altogether, these results suggests dual therapeutic role of Notch signaling pathway by inhibiting both key pathogenic cells in liver fibrosis i.e. fibrogenic cells (HSCs) and inflammatory M1 macrophages.

## Materials and Methods

### Human Liver Specimens

Human liver specimens were obtained from autopsy obtained from patients suffering from liver cirrhosis and were anonymously provided by the Department of Pathology, Laboratory Pathology East Netherlands (LabPON), Enschede, The Netherlands. Ethical approvals were approved by the local Medical Ethical Committee at LabPON. The use of human tissues for this study was approved by the Local Ethics Committee, University of Twente. All the experiments involving human tissues were performed in accordance with institutional guidelines and regulations. All the experimental protocols were approved by institutional committee at University of Twente. All the samples collected were from human subjects who gave informed consent for their tissues to be used for research purposes.

### Cell Lines

Mouse NIH3T3 fibroblasts and murine RAW264.7 macrophages were obtained from the American Type Culture Collection (ATCC, Manassas, VA, USA). Human hepatic stellate cells (LX2) were kindly provided by Prof. Scott Friedman (Mount Sinai Hospital, New York). RAW macrophages and 3T3 were cultured in Roswell Park Memorial Institute (RPMI) 1640 medium (Lonza, Verviers, Belgium) and Dulbecco’s modified Eagle’s (DMEM) medium (Lonza) respectively supplemented with 2 mM L-glutamine (Sigma, St. Louis, MO), 10% fetal bovine serum (FBS, Lonza) and antibiotics (50 U/ml Penicillin and 50 μg/ml streptomycin, Sigma). Human LX2 were cultured in DMEM-Glutamax (Invitrogen, Carlsbad, CA) supplemented with 10% FBS and antibiotics (50 U/ml Penicillin and 50 μg/ml streptomycin, Sigma).

### *In vitro* effects of Avagacestat in human HSC and mouse fibroblasts

Cells were seeded in 24 well plates (30,000 cells/well for stainings) and 12 well plates (80,000 cells/well for quantitative PCR analysis) and cultured overnight. To assess the effects on fibrotic parameters, cells were starved for 24 h and then incubated with starvation medium alone, Avagacestat (Selleckchem Boston, USA; 5 μM and 10 μM for stainings and 10 μM for quantitative PCR) and 5 ng/ml of human recombinant TGFβ1 (Roche, Mannheim, Germany) for 24 h. Cells (24 well plates) were then fixed with chilled acetone:methanol (1:1) for 20 min, dried and stained for collagen I, α-SMA and vimentin (antibodies and dilutions are summarized in supplementary Table 1). In addition, cells (12 well plates) were lysed with RNA lysis buffer constituted with β-mercaptoethanol (Sigma) to perform quantitative real time PCR analyses for fibrotic parameters (Collagen 1α1, α-SMA and vimentin) and Notch pathway genes (Notch-1, -2 and -3, Dll-1 and -4, Jag1 and Hes1). Stainings and quantitative PCR analysis was performed on three independent experiments.

To assess effects on cell viability, cells plated in 96 well plates were serum-starved for 24 h and incubated with different concentrations of Avagacestat (0.5, 1, 5 and 10 uM) for 24 h. Cell viability assay was performed using Alamar Blue reagent (Invitrogen). The results are represented as % cell viability normalized to untreated control cells (at 100%). All measurements were performed in triplicates in three independent experiments.

### 3D collagen I gel contraction assay

A collagen gel contraction assay was performed as described previously^41^ with minor modifications. We used this assay to examine the inhibitory effect of Avagacestat on the contractile activity of LX2 cells. A collagen suspension (5 ml) containing 3.0 ml Collagen G1 (5 mg/ml, Matrix biosciences, Morlenbach, Germany), 0.5 ml 10x M199 medium (Sigma), 85 ul 1N NaOH (Sigma) and sterile water was mixed with 1.0 ml (2 × 10^6^ cells) human LX2 cells. Collagen gel-cells suspension (0.6 ml/well) was plated in a 24‐well culture plate and allowed to polymerize for 1 h at 37 °C. Once polymerized, 1 ml of 0.5% serum containing medium was added with or without TGFβ (5 ng/ml) together with 10 μM Avagacestat or PBS followed by detachment of the gels from the culture wells. Digital images were made at 0, 24, 48 and 72 h using a Nikon Coolpix digital camera (Nikon, Mississauga, ON, Canada). Measurement of collagen gel diameter at the indicated time points was performed using Image J imaging software (NIH, Bethesda, MD). Gels were measured by tracing around the edges of the gel disk and normalized with their respective well size in each image. Gel contraction experiments were performed in duplicates in three independent experiments.

### *In vitro* effects of Avagacestat in differentiated mouse RAW Macrophages

For differentiation of RAW macrophages, cells were plated (1 × 10^6^ cells/well) in 12 well plates and cultured overnight. The cells were then incubated with LPS (10 ng/ml) and IFNγ (10 ng/ml) for M1 differentiation, and IL-4 (10 ng/ml) and IL-13 (10 ng/ml) for M2 differentiation for 24 h. To study the effect of Notch inhibitor, 10 μM Avagacestat was added with M1- and M2-differentiating cytokines and incubated for 24 h. Cells were then lysed with RNA lysis buffer and samples were analyzed for M1-specific genes (IL-1β, NOS2 and IL-6) and M2-specific genes (MRC1 and Arginase 1) by quantitative PCR. All the experiments were performed in three independent experiments.

### Co-culture and conditioned medium studies

#### For co-culture studies

3T3 fibroblasts and RAW macrophages were co-cultured together or alone as monocultures in complete DMEM medium supplemented with antibiotics and 10% FBS. After overnight culture, cells were serum-starved for 24 h. Thereafter, co-cultured cells were incubated with TGFβ (5 ng/ml) and M1 cytokines (10 ng/ml LPS and 10 ng/ml IFNγ) with and without Avagacestat (10 μM) for 24 h. Then, medium was collected and nitric oxide release assay was performed to study 3T3-driven activation of M1 macrophages.

#### For conditioned medium studies

3T3 fibroblasts and RAW macrophages were plated in complete DMEM and RPMI medium respectively in 12 well plates and cultured overnight. Then cells were serum-starved for 24 h. Then 3T3 fibroblasts were incubated with or without TGFβ (5 ng/ml), with and without Avagacestat (10 μM). RAW macrophages were incubated with or without M1 cytokines [LPS (10 ng/ml) and IFNγ (10 ng/ml)], with and without Avagacestat (10 μM). After 24 h, incubated cells were washed and fresh starved medium was added. After 24 h, conditioned medium was collected and stored at −70 °C until use. 3T3 conditioned medium (from different conditions) was added on RAW macrophages and M1-differentiated macrophages with an equal volume of fresh medium to avoid nutrient depletion effects. RAW macrophages conditioned medium (from different conditions) with equal volume of fresh medium was added on 3T3 fibroblasts. After 24 h incubation with the conditioned medium, medium was collected for nitric oxide release assay and cells were lysed for quantitative PCR analysis. All the co-culture and conditioned medium studies were performed in three independent experiments.

### Nitric Oxide (NO) release bioassay

The effect of Avagacestat on M1 macrophages was assessed by measuring the inhibition in nitrite NO_2_ release, a stable NO metabolite produced by M1 macrophages. RAW cells (2 × 10^5^ cells/200 ul/well) seeded in 96-well plates were cultured with IFNγ (10 ng/ml) and LPS (10 ng/ml) together with different concentrations of Notch inhibitor Avagacestat (0, 0.5, 1, 5 and 10 μM). After 24 h, 100 μl culture supernatant were added to 100 μl of Griess reagent (1% sulfanilamide; 0.1% naphthylethylendiamine dihydrochloride; 3% phosphoric acid) and absorbance at 540 nm was measured with a microplate reader.

### Animal Experiments

All the animal experiments in this study were performed in strict accordance with the guidelines and regulations for the Care and Use of Laboratory Animals, University of Twente, The Netherlands. The protocols were approved by the Institutional Animal Ethics Committee of the University of Twente, The Netherlands. Male 6- to 8-week old C57BL/6 mice were purchased from Harlan (Zeist, Netherlands) and kept at 12 h light/12 h dark cycles with ad libitum normal diet.

### CCl_4_-induced 4 weeks and 8 weeks liver fibrosis mouse models

Male Balb/c mice (6–10 weeks old; n = 5 per group) were treated with increasing concentration of carbon tetrachloride (CCl_4_) (week 1: 0.5 ml/kg; week 2: 0.8 ml/kg and week 3–8: 1 ml/kg prepared in olive oil) twice weekly by intra-peritoneal injections for 4 or 8 weeks. Olive oil treated non-fibrotic mice are used as controls. The mice were sacrificed at week 4 and week 8 by cervical dislocation and livers were collected for subsequent analysis.

### CCl_4_-induced acute liver injury mouse model

To study the inhibitory effect of Notch inhibitor, male C57BL/6 mice (20–22 g) were treated with a single intraperitoneal injection of CCl_4_ (1 ml/kg in olive oil) at day 1.Olive-oil treated mice were used as controls. At day 2 and day 3, CCl_4_-treated mice intraperitoneally received 10 mg/kg Avagacestat prepared in 1% DMSO (Sigma) and 5% β-hydroxycyclodextrin (Sigma) or vehicle treatment (1%DMSO/5%β-hydroxycyclodextrin/PBS) (n = 5 per group). At day 4, all mice were sacrificed and livers were harvested for subsequent analysis.

Progressive 4 and 8 weeks model has been established in Balb/c mice while C57BL/6 mouse strain was used for acute liver injury model. From our experience, C57BL/6 mice have high mortality, following 4–8 weeks CCl_4_ treatment and leads to multi-organ fibrosis. While CCl_4_ administration in Balb/c mice, leads to slow and progressive fibrosis and hence are not suitable for acute liver injury model. Therefore, we have established acute liver injury model in C57BL/6 to have early liver fibrogenesis and Balb/c for progressive (advanced) fibrosis. The difference between these mice strains lies in the differences in Th1 and Th2 responses^42^. Th1-dominance response in C57BL/6 strain and heightened Th2-responses in Balb/c mice determine the severity of liver fibrosis in these mice strains.

### Immunohistochemistry and Immunoflourescence

Liver tissues were harvested and transferred to Tissue-Tek OCT embedding medium (Sakura Finetek, Torrance, CA), and snap-frozen in 2-methyl butane chilled in a dry ice. Cryosections (4 μm) were cut using a Leica CM 3050 cryostat (Leica Microsystems, Nussloch, Germany). The sections were air-dried and fixed with acetone for 10 min. Cells or tissue sections were rehydrated with PBS and incubated with the primary antibody in appropriate dilution (refer to supplementary Table 1) for 1 h at room temperature. Cells or sections were then incubated with horseradish peroxidase (HRP)-conjugated secondary antibody for 1 h at room temperature. Then incubated with HRP-conjugated tertiary antibody or donkey anti-goat Alexa 594 labeled tertiary antibody (Life Technologies, Gaithersburg, Md) for 1 h, after which these were washed thrice with 1x PBS. Thereafter, peroxidase activity was developed using AEC (3-amino-9-ethyl carbazole) substrate kit (Life Technologies) for 20 min and nuclei were counterstained with hematoxylin (Fluka Chemie, Buchs, Switzerland). For tissue sections, endogenous peroxidase activity was blocked by 3% H_2_O_2_ prepared in methanol. Cells or sections were mounted with Aquatex mounting medium (Merck, Darmstadt, Germany). The staining was visualized and the images were captured using light microscopy (Nikon eclipse E600 microscope, Nikon, Tokyo, Japan). For immunofluorescence, sections were mounted with DAPI containing mounting medium and examined using Hamamatsu NanoZoomer Digital slide scanner 2.0HT (Hamamatsu Photonics, Bridgewater NJ).

### Quantitative histological analysis

For quantitation, stained sections were scanned at high resolution using Hamamatsu NanoZoomer Digital slide scanner 2.0HT (Hamamatsu Photonics). High resolution scans were viewed using NanoZoomer Digital Pathology (NDP2.0) viewer software (Hamamatsu Photonics). About 20 images (100x) of each entire section (from NDP) were imported into NIH ImageJ software (NIH, Bethesda, MD) and were analyzed quantitatively at a fixed threshold.

### RNA extraction, reverse transcription and quantitative real time PCR

Total RNA from cells and liver tissues was isolated using GenElute Total RNA Miniprep Kit (Sigma) and SV total RNA isolation system (Promega Corporation, WI, USA) respectively according to manufacturer’s instructions. The RNA concentration was quantitated by a UV spectrophotometer (NanoDrop Technologies, Wilmington, DE). Total RNA (1 μg) was reverse-transcribed using iScript cDNA Synthesis Kit (Bio-Rad, Hercules, CA). All the primers were purchased from Sigma-Genosys (Haverhill, UK). Real-time PCR was performed using 2x SensiMix SYBR and Fluorescein Kit (Bioline, QT615-05, Luckenwalde, Germany), 20 ng cDNA and pre-tested gene-specific primer sets (listed in supplementary Table 2 and 3). The cycling conditions for the BioRad CFX384 Real-Time PCR detection system were 95 °C for 10 min, 40 cycles of 95 °C/15 sec, 58 °C/15 sec and 72 °C/15 sec. Finally, cycle threshold (Ct) values were normalized to reference gene GAPDH and fold changes in expression were calculated using the 2^−ΔΔCt^ method.

### Western blot analysis

Liver tissues were homogenized in cold RIPA buffer (50 mM Tris–HCl, 150 mM NaCl, 0.1% SDS, 0.1% Igepal in 0.5% sodium deoxycholate with 1 tablet of protease inhibitor cocktail and 1 tablet of phosphatase inhibitor (Roche Diagnostics, Mannheim, Germany) in 10 ml) on ice with a tissue homogenizer and the lysates were centrifuged at 12,000  rpm for 1 h at 4 °C. The supernatants were stored at −70 °C until use. The samples were boiled in standard protein sample buffer (Life Technologies) and subjected to SDS-PAGE with 10% Tris-glycine gels (Life Technologies) followed by protein transfer onto PVDF membrane. The membranes were developed according to the standard protocols using primary and secondary antibodies as mentioned in supplementary Table 4. The bands were visualized using ECL detection reagent (Perkin Elmer Inc., Waltham, MA) and photographed using FluorChem M Imaging System (ProteinSimple, Alpha Innotech, San Leandro CA). Intensity of individual bands was quantified using ImageJ densitometry software, and expressed in % relative to β-actin.

### Statistical analyses

All the data are presented as mean ± standard error of the mean (SEM). The graphs and statistical analyses were performed using GraphPad Prism version 5.02 (GraphPad Prism Software, Inc., La Jolla, CA, USA). Multiple comparisons between different groups were performed by one-way analysis of variance (ANOVA) with Bonferroni post-test. The differences were considered significant at p < 0.05.

## Additional Information

**How to cite this article**: Bansal, R. *et al.* The interplay of the Notch signaling in hepatic stellate cells and macrophages determines the fate of liver fibrogenesis. *Sci. Rep.* **5**, 18272; doi: 10.1038/srep18272 (2015).

## Supplementary Material

Supplementary Information
