# Supplementary material for: The interplay of the Notch signaling in hepatic stellate cells and macrophages determines the fate of liver fibrogenesis
Source: Sci Rep. 2015 Dec 14;5:18272. doi: 10.1038/srep18272 (PMC4677309; doi:10.1038/srep18272)
Supplement: Supplementary Information [file srep18272-s1.pdf]

The interplay of the Notch signaling in hepatic stellate cells and macrophages determines the fate of liver fibrogenesis

Ruchi Bansal[1*]; Joop van Baarlen[2]; Gert Storm[1,3]; Jai Prakash[1]

Supplementary Table 1: Antibodies used for the immunohistochemistry

**Primary Antibody**	**Source**	**Dilution**
Polyclonal rabbit α-SMA	Abcam	1:100
Polyclonal goat anti-collagen I	Southern Biotech	1:100
Polyclonal goat anti-desmin	Santa Cruz	1:100
Polyclonal rat anti-F4/80 (MCA497)	AbD Serotec	1:100
Polyclonal goat anti-vimentin (sc-7557)	Santa Cruz	1:100
Polyclonal rabbit anti-Notch1 (NBP1-78292)	Novus Biologicals	1:100
Polyclonal rabbit anti-Notch3 (M134, sc-5593)	Santa Cruz	1:100
Monoclonal rat MHC-II (ER-TR3, sc-59318)	Santa Cruz	1:100
Polyclonal goat anti-chitinase 3-like 3/ECF-L (YM1)	R & D systems	1:100
**Secondary Antibody**	**Source**	**Dilution**
Polyclonal Goat anti-rabbit IgG	DAKO	1:100
Polyclonal Rabbit anti-rat IgG	DAKO	1:100
Polyclonal Rabbit anti-Goat IgG	DAKO	1:100

Supplementary Table 2: Sequence of the mouse primers used for quantitative real-time PCR

Gene	Forward primer sequence (5'-3')	Reverse primer sequence (5'-3')
Arginase I	GTGAAGAACCCACGGTCTGT	CTGGTTGTCAGGGGAGTGTT
Collagen I	TGACTGGAAGAGCGGAGAGT	ATCCATCGGTCATGCTCTCT
Desmin	ATGCAGCCACTCTAGCTCGT	CTCATACTGAGCCCGGATGT
Dll1	TCTAGAACACTCTGGGAGCGG	CTATTAGGAAGCGGCGGTCT
Dll4	TATACCTGCACCTGTCTCCCA	TTACAGCTGCCACCATTTCG
GAPDH	ACAGTCCATGCCATCACTGC	GATCCACGACGGACACATTG
Hes1	GCGGAATCCCCTGTCTACC	GTCTTAGGGCTACTTAGTGATCG
IL-1β	GCCAAGACAGGTCGCTCAGGG	CCCCCACACGTTGACAGCTAGG
IL-6	TGATGCTGGTGACAACCACGGC	TAAGCCTCCGACTTGTGAAGTGGTA
NOS2	GGTGAAGGGACTGAGCTGTT	GCTACTCCGTGGAGTGAACAA
Jag1	ACTGGTGTGTAAGGAAGCGG	CGCACATTGTTGGTGGTGTT
MRC1	GGGACGTTTCGGTGGACTGTGG	TTGTGGGCTCTGGTGGGCGA
Notch1	TGTGGCTTCCTTCTACTGCG	CTTTGCCGTTGACAGGGTTG
Notch2	AGCTTGGGCAGGTTACATCC	TGGACATGTGCTTCCCTTCC
Notch3	GACTGCTCACTGAACGTGGA	CACACCGGCTGTTGTTGAAG
SOX9	GTGCAAGCTGGCAAAGTTGA	TGCTCAGTTCACCGATGTCC
Vimentin	TCCAGAGAGAGGAAGCCGAA	AAGGTCAAGACGTGCCAGAG
α-SMA	ACTACTGCCGAGCGTGAGAT	CCAATGAAAGATGGCTGGAA

Supplementary Table 3: Sequence of the human primers used for quantitative real-time PCR

Gene	Forward primer sequence (5'-3')	Reverse primer sequence (5'-3')
Collagen I	GTACTGGATTGACCCCAACC	CGCCATACTCGAACTGGAAT
Desmin	GCGGGTTTCGGCTCTAAGG	AGAAACTCCTGGTTCACCGC
Dll1	GAGGCACTGTGACGACAACG	GCACACTCGCACACATAGCG
Dll4	AAGGCTGCGCTACTCTTACC	GTCATTGCGCTTCTTGCACA
GAPDH	TCCAAAATCAAGTGGGGCGA	TGATGACCCTTTTGGCTCCC
Hes1	CTGAGCACAGACCCAAGTGT	GAGTGCGCACCTCGGTATTA
Jag1	TGAGGCCGTTGCTGACTTAG	TGAGATGCGGCACTCGATTT
Notch1	GAATGGTCAATGCGAGTGGC	CCGCAGAGGGTTGTATTGGT
Notch2	AGAATCAGCCCTGCCAGAAT	CAATGCCCTGGATGGAAAATGG
Notch3	CATGGTATCTGCACCAACCT	TTGATGTCCTGATCGCAGGAAG
Vimentin	AAATGGCTCGTCACCTTCGT	CAGCTTCCTGTAGGTGGCAA
α-SMA	CCCCATCTATGAGGGCTATG	CAGTGGCCATCTCATTTTCA

Supplementary Table 4: Antibodies used for western blot analysis

Primary Antibody	Source	Dilution
Polyclonal rabbit anti-Notch1 (NBP1-78292)	Novus Biologicals	1:250
Polyclonal rabbit anti-Notch3 (M134, sc-5593)	Santa Cruz	1:250
Monclonal mouse β-actin antibody	Sigma Aldrich	1:5000
**Secondary Antibody**	**Source**	**Dilution**
Polyclonal Goat anti-rabbit IgG	DAKO	1:2000
Polyclonal goat anti-mouse IgG	DAKO	1:1000
Polyclonal Rabbit anti-Goat IgG	DAKO	1:2000
